# The Role of Temporal Information in Perisaccadic Mislocalization

**DOI:** 10.1371/journal.pone.0134081

**Published:** 2015-09-09

**Authors:** Maria Matziridi, Eli Brenner, Jeroen B. J. Smeets

**Affiliations:** Department of Human Movement Sciences, Research Institute MOVE, VU University, Amsterdam, The Netherlands; State University of New York Downstate Medical Center, UNITED STATES

## Abstract

In dynamic environments, it is crucial to accurately consider the timing of information. For instance, during saccades the eyes rotate so fast that even small temporal errors in relating retinal stimulation by flashed stimuli to extra-retinal information about the eyes’ orientations will give rise to substantial errors in where the stimuli are judged to be. If spatial localization involves judging the eyes’ orientations at the estimated time of the flash, we should be able to manipulate the pattern of mislocalization by altering the estimated time of the flash. We reasoned that if we presented a relevant flash within a short rapid sequence of irrelevant flashes, participants’ estimates of when the relevant flash was presented might be shifted towards the centre of the sequence. In a first experiment, we presented five bars at different positions around the time of a saccade. Four of the bars were black. Either the second or the fourth bar in the sequence was red. The task was to localize the red bar. We found that when the red bar was presented second in the sequence, it was judged to be further in the direction of the saccade than when it was presented fourth in the sequence. Could this be because the red bar was processed faster when more black bars preceded it? In a second experiment, a red bar was either presented alone or followed by two black bars. When two black bars followed it, it was judged to be further in the direction of the saccade. We conclude that the spatial localization of flashed stimuli involves judging the eye orientation at the estimated time of the flash.

## Introduction

Localizing something seems to be one of the easiest tasks that our visual system can perform, especially if this ‘something’ is a simple spot of light on a black screen, so that there is no difficulty segregating it from the background. However, Matin and Pearce [1] discovered that people make systematic errors when localizing flashes that are presented around the time of saccades (also see [2–13]). Two components of these errors have been identified: a shift in the flashes’ apparent locations in the direction of the saccade [1–3,5,9,14–16] and a spatial compression of the flashes’ apparent locations toward the saccade target location [6–8,12,17,18] or towards the position that is fixated after the saccade [13,19,20].

The shift is explained by a temporal mismatch between signals arising from stimulation of the retina and signals related to the orientation of the eye [5,9]. The cause of the compression has been debated. Some attribute it to a mechanism for remapping the visual scene across saccades [21], but it has also been suggested that the compression arises from a combination of temporal uncertainty with a bias to believe that what one has seen was where one was looking [22,23].

The two explanations for compression differ with respect to when retinal and oculomotor signals are combined. According to the remapping explanation, the time course of the combination only depends on the eye movement, so it influences all items on the retina before one has selected the item of interest (the flash). In accordance with this suggestion, anticipatory shifts of neurons’ receptive fields have been observed in many cortical areas [24–26]. According to the other explanation, vision is a more active process [27]. The item of interest (the flash) first has to be detected. The relevant eye orientation is subsequently estimated in order to localize the detected item. A similar explanation has been proposed in relation to moving targets to account for flash lag [28]. In this more active explanation, the localization depends critically on when the flash is considered to have occurred. Misjudging this moment can result in the flash being mislocalized relative to the background [20,29].

In support of the more active explanation, Maij et al. [12] found that presenting a tone at different moments with respect to the time of a flash shifted the pattern of mislocalization of flashes presented near the times of saccades. They argued that the average mislocalization was consistent with the time of the flash being a weighted average of the judged times of the flash and the tone, considering the probability of the two arising from a single event. This conclusion is based in the assumption that visual and auditory stimuli are combined when judging time, just as they are when judging position [30–32]. However, Binda et al. [33] could only reproduce this effect when using flashes that were hard to detect. They interpreted the small effect of the tone on the flash in Maij et al.’s experiment in terms of auditory cueing of the appearance of the flash. Misjudging timing due to auditory cueing and misjudging timing by combining sound and vision to judge the time of the flash are both consistent with perisaccadic mislocalization being caused by misjudging the moment of the flash before combining its retinal location with oculomotor information.

If errors in judging the time of the flash are (partly) responsible for the reported mislocalization of flashes presented around the time of saccades, we should be able to manipulate the pattern of mislocalization by altering the perceived time of the flash in other ways than with tones. To see whether this is the case, we designed two experiments. In the first experiment we presented a rapid sequence of five bars (one red and four black; 10 ms intervals) at different positions around the time of a saccade. The task was to localize the red bar that was always either the second or the fourth in the sequence. In the second experiment we either presented a single red bar or a red bar followed by two black bars (at 10 ms intervals; one to the right and one to the left of the red bar). Again, the task was to localize the red bar.

Since the resolution of human temporal order judgments is probably too poor to accurately identify the red bar’s temporal position in the sequence [22,34–38], we expect participants’ judgments of the red bar’s timing to be shifted towards the centre of the sequence; the time of the presentation of the third bar in the first experiment and the time of the presentation of the second bar when there are three bars in the second experiment. If the asymmetrically timed, irrelevant black bars influence the estimated timing of the relevant red bar, they will affect the judgments of its spatial location: when the red bar is presented early in the sequence (in the first experiment), or is followed by two black bars (in the second experiment), it will be perceived as having occurred later in time, and thus be judged to be further in the direction of the saccade, than when it is presented late in the sequence (in the first experiment) or alone (in the second experiment). Thus, we expect to see a shift between the perceived positions in the two conditions within each experiment. Since we attribute compression to temporal uncertainty, we do not expect the amount of compression to differ between the conditions unless the timing of the irrelevant black bars influences the precision with which participants can judge when or where the red flash occurred.

We conducted one pilot and two main experiments. We conducted the pilot experiment to verify that the resolution of human temporal order judgments is indeed too poor to accurately identify the presentation order of the five bars. The rationale behind experiment 1 was to present five bars on every trial, so that participants were not aware of there being two conditions. However, when the red bar was presented second, it was preceded by one black bar, 10 ms earlier, whereas when the red bar was presented fourth, it was preceded by three black bars, the first of which was presented 30 ms earlier. Perhaps having more preceding black bars speeds up the processing of the red bar. To exclude any contribution of the preceding black bar(s) on the localization of the red bar, we conducted experiment 2, in which the red bar was always presented first.

## Methods

All the participants that took part in the present study were naive with respect to the aim of the experiment in question, had normal or corrected-to-normal vision and gave written informed consent prior to participation. The study is part of a research program that has been approved by the ethics committee of the Faculty of Human Movement Sciences (ECB 2006–02).

### Pilot experiment

#### Participants

Three right-handed participants (age 29±1 years; female) volunteered to take part in the pilot experiment.

#### Apparatus and experimental setup

The purpose of the pilot experiment was to evaluate human temporal order judgments for the stimuli used in Experiment 1. The apparatus, setup, stimuli and conditions were therefore designed to be as close as possible to those used in Experiment 1 (see below). The experiment was conducted in a normally illuminated room (fluorescent lamps). The participant sat in front of a touch screen (EloTouch CRT 19”, 800 x 600 pixels, 36 x 27 cm, 100 Hz) on which visual stimuli were presented by a computer using the Psychophysics Toolbox [39]. The touch screen was calibrated using the standard nine-point calibration provided by Elo-Touch. A chin-rest was placed in front of the touch screen to keep the participant’s head fixed at a viewing distance of 57.3 cm. At this viewing distance, one cm equals one degree of visual angle.

#### Stimuli and conditions

The stimuli consisted of a sequence of five flashed vertical bars (0.35 cm x 1.5 cm). Four of the bars were black (9 cd/m^2^) and one was red (29 cd/m^2^; CIE_xy_ = 0.54, 0.35), all on a white background (125 cd/m^2^; CIE_xy_ = 0.28, 0.32). The five bars were presented near the centre of the screen, separated horizontally by 1.4 cm. Their centres were either aligned with the centre of the screen, or either 0.75 or 1.5 cm higher or lower.

The bars were not presented simultaneously, but one each on five consecutive frames (i.e. at 10 ms intervals). The red bar was presented on one of these frames; a black bar was presented on the other four frames. Independent of its timing, the red bar could be at any of the five possible horizontal locations. The four black bars were randomly distributed over the other four possible horizontal locations. The vertical locations of all five bars were chosen at random from the five possible locations, with the restriction that two successive bars were never at the same vertical location. The different vertical locations were introduced for two reasons: to make it easier for the participants to identify the independent bars, and to be able to check in Experiment 1 that it was the timing of the red bar that was misjudged, rather than its identity.

Not considering the different horizontal and vertical locations of the red and the black bars, we distinguish five conditions, each with the red bar at a different time in the sequence. Each participant performed one session of 400 trials; 80 for each of the five conditions. The trials were presented in random order, with the restriction that the same condition was never presented on successive trials. There was a short break half way through the session.

#### Procedure

A trial started with the sequence of five bars being presented on the screen. After the presentation of the five bars, five numbers (1–5) appeared 10 cm below the screen centre. The distance between the centre of the numbers was 7 cm. The participants were asked to indicate in which place in the temporal sequence the red bar had been presented by touching the screen with the index finger of their dominant hand at the location of the corresponding number. Once the screen had been touched, a new trial started with a new sequence of five bars. Participants were free to look where they pleased during the whole trial.

#### Data analysis

*Touch position*: The number near the first position at which the finger touched the screen was considered to indicate the participant’s judgment about the temporal position of the red bar in the sequence. We rejected touch locations that were more than 2 cm from the centre of a number (about 0.6% of the trials).

*Evaluating performance*: We determined the percentage of correctly indicated times of the red bar in the sequence. We also determined the percentages of trials in which participants indicated times one, two, three or four places earlier or later in the sequence. We compared these percentages with what one would expect if subjects were responding at random.

### Experiment 1

#### Participants

Nine participants (age 28±2 years; six female) volunteered to take part in this experiment. Eight of them were right-handed and one left-handed. None of them took part in the pilot experiment.

#### Apparatus and experimental setup

The apparatus and experimental setup was similar to that used for the pilot experiment. In addition, eye movements were recorded with an Eyelink II eye tracker (SR Research Ltd.) using the Eyelink toolbox [40]. This system records eye position with a spatial resolution of 0.2° and a temporal resolution of 500 Hz. The recording of the eye movements was calibrated using the standard nine-point calibration procedure of the Eyelink II. To determine the precise timing of stimulus presentation on the screen in relation to the recorded eye movement, a 2 cm white dot was presented on a black square (2 x 2 cm) in the lower right corner of the screen. This dot was presented on the same frame as the flashed red bar. It was not visible to the participant, but a photo-diode attached to the lower right corner of the screen measured the light from this dot and sent a signal to the parallel port of the computer as soon as the dot appeared. This signal was recorded in the Eyelink data file, which allowed us to later know precisely when the red bar occurred in relation to the eye movement [41]. The red bar was flashed at different places on the screen, so its timing relative to that of the white dot varied by a few milliseconds. No corrections were made for these timing differences between red bars presented at different places on the screen.

#### Stimuli and conditions

**Fig 1:**
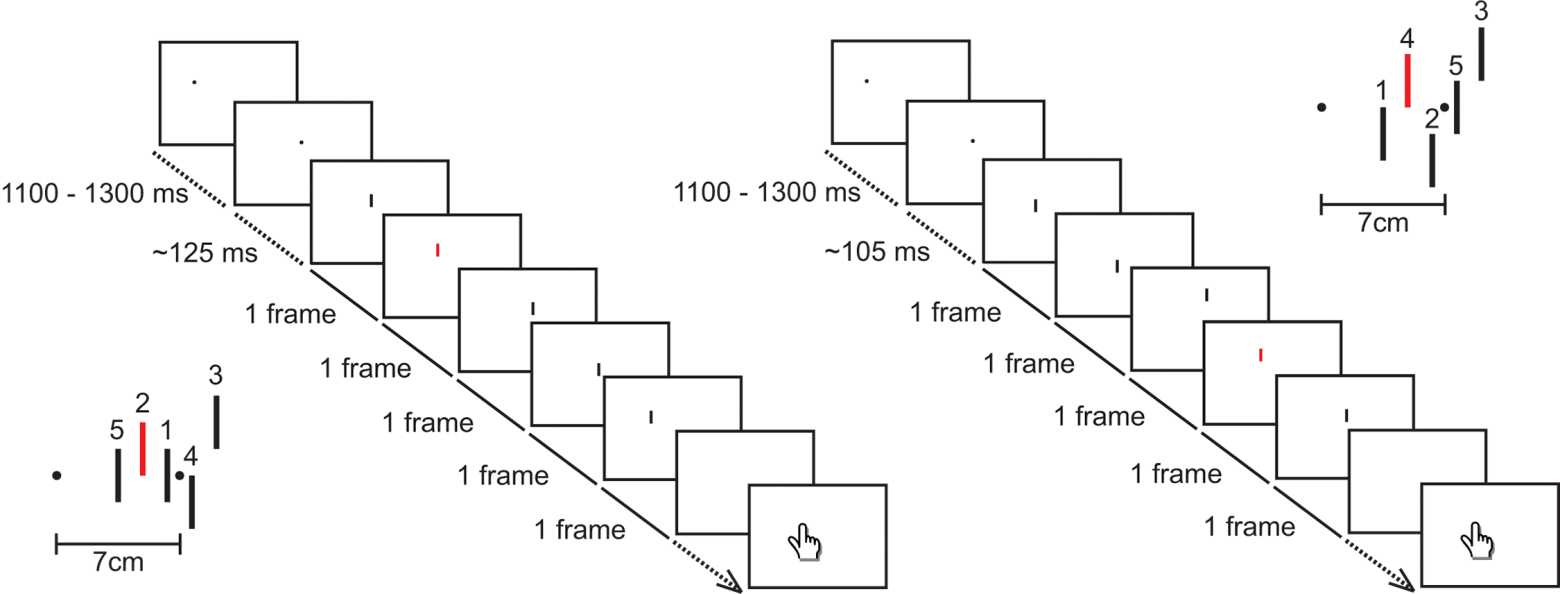
Schematic overview of two example trials in Experiment 1. A black fixation dot appears on the screen. After a random interval (range 1100–1300 ms), it is replaced by a black target dot that appears on the screen 7 cm to the right of the fixation dot. Around the time at which the participant makes a saccade to the target dot, a rapid sequence of five bars is presented (one red and four black; 10 ms intervals; one frame each at different locations). The red bar was presented second (left part of the figure) or fourth (right part of the figure) in order. In both these example trials, the red bar was presented about 135 ms after the presentation of the target dot. The target dot had always disappeared by the time the saccade ended. Participants had to indicate where they had seen the red bar, by touching its location with their index finger. The insets show the spatial configurations of these example trials, with numbers indicating orders of presentation.

The stimuli consisted of a black (9 cd/m^2^) fixation dot (0.44 cm diameter), a black target dot (0.44 cm diameter) and five flashed vertical bars (four black and one red; identical to the ones of the pilot experiment), all on a white background (Fig 1). In each trial, one fixation dot, one target dot and five bars were presented on the screen. The fixation dot was presented randomly at one of 20 possible locations on the screen. The target dot was always presented 7 cm to the right of the fixation dot. The five bars were always flashed 3.5, 4.9, 6.3, 7.7 and 9.1 cm to the right of the fixation dot, which is at 50%, 70%, 90%, 110% and 130% of the distance between the fixation and the target dot. The relative horizontal, vertical and temporal positions of the five bars were identical to the ones of the pilot experiment. The only difference is that the red bar was always presented second or fourth.

Not considering the variations in the position of the fixation dot, in the vertical location of the red bar, or in the location of the black bars, there were 10 conditions: 2 red bar orders x 5 red bar horizontal locations. Each session consisted of 400 trials; 40 for each of these conditions. The trials were presented in random order within a session, with the restriction that the same condition was never presented on successive trials. The fixation dot was also never presented at the same location on successive trials. There was a short break half way through the session. All participants performed at least 4 sessions. We added additional sessions if we were uncertain about the value for the mislocalization for any instant between 30 ms before and 60 ms after the saccade onset. This was the case if it was possible to find an 8 ms time window (twice the standard deviation of the Gaussian used for smoothing; see *Mislocalization* *section of the data analysis*) with fewer than 3 successful trials. On average, participants had to perform five sessions to yield reliable data throughout the relevant time-window.

#### Procedure

A trial started with a fixation dot appearing on the screen (Fig 1). Participants had to fixate the dot. After a random interval of 1100–1300 ms, the fixation dot disappeared and the target dot appeared on the screen. Participants were asked to make a saccade from the fixation dot to the target dot as soon as the target dot appeared on the screen. To be able to present the bars near the moment of the saccade, we predicted the saccade onset for each new trial on the basis of the average saccadic latency (the time between the presentation of the target dot and the start of the saccade) on previous trials [12]. The red bar, that was always presented second or fourth, was presented for one frame near the predicted time of the saccade onset, at one of the five locations. The other bars flashed for one frame each at the other four horizontal locations. The target dot disappeared at the frame of the first flash (60–160 ms after target onset). The participants were asked to touch the screen with the index finger of their dominant hand at the location at which they saw the red bar. By the time they touched the screen all stimuli had disappeared. If participants had not seen the bar for some reason, they could indicate having missed it by touching the bottom of the screen. Once the screen had been touched, a new trial started with a new fixation dot appearing at a new position on the screen.

Note that when the red bar was presented second in order, the timing of the whole sequence extended from 10 ms before to 30 ms after the presentation of the red bar, whereas when the red bar was presented fourth in order, the timing of the whole sequence extended from 30 ms before to 10 ms after the presentation of the red bar. Thus, when the red bar was presented second in order, the mean timing of the sequence as a whole was 10 ms later than the presentation of the red bar, whereas when the red bar was presented fourth in order, the mean timing of the sequence was 10 ms earlier than the presentation of the red bar. The spatial locations of the five bars were the same on all trials, and we compare localization when the red bar was presented at the same time relative to the saccade onset (irrespective of whether it was second or fourth in order).

#### Data analysis

*Gaze and touch position*: The Eyelink’s gaze position data of the right eye were used to determine characteristics of the primary saccades (the first saccades occurring after the target dot appeared on the screen). The first of two consecutive sampling intervals for which the tangential velocity of the eye movement exceeded 35°/s was considered to be the saccade onset, and the first sample after that at which the velocity returned below this value was considered to be the saccade end. The first position at which the finger touched the screen was considered to be the perceived position of the red bar, or, if the bottom of the screen was touched, an indication that the participant had not seen the red bar.

Trials were discarded if there was no saccade between 100 ms before and 100 ms after the time of the presentation of the red bar (wrong timing; about 7% of the trials), if the length of the saccade was less than 50% or more than 150% of the 7 cm distance between the fixation and the target dot (wrong amplitude; about 7% of the trials), if the direction of the saccade deviated by more than 22.5° from a movement to the right (wrong direction; about 1% of the trials), if the saccadic reaction time was less than 85 ms or more than 300 ms (wrong latency; about 6% of the trials) or if the touched location differed by more than 7 cm horizontally or by more than 3 cm vertically from the centre of the bar (wrong or no localization; mainly trials in which participants touched the bottom of the screen to indicate that they had not seen the red bar; about 3% of the trials).

*Mislocalization*: We were mainly interested in localization in the direction of the target dot. We therefore defined the perceived position as the horizontal distance from the fixation dot to the touched location. As the saccade latency varied from trial to trial, the bars were presented at various times relative to saccade onset. To draw a smooth curve through the data for each condition (i.e. each bar order and bar horizontal location), we averaged the perceived positions with weights based on a (moving) Gaussian window (σ = 4 ms). We did so for each participant and condition and then averaged these values at each moment across participants. We will refer to the resulting curves as mislocalization curves.

Our prediction for the difference between the conditions is that the curves for trials in which the red bar is presented early in the sequence will be above those for trials in which the red bar is presented late in the sequence. We will test whether this is the case for the main time of interest: during the saccade. To do so, we will average the differences between the mislocalization curves for the conditions in which the red bar was presented second and the corresponding mislocalization curves for the conditions in which the red bar was presented fourth (i.e. the distance between corresponding curves) across the duration of the saccade and the five bar locations for each subject, and use a one-tailed, one-sample t-test to check whether this difference is positive, i.e. whether the order of the flashes has the predicted systematic shift of perceived locations.

To check whether participants failed to isolate the relevant bar from the irrelevant ones spatially (as presumably occurred in [42]), we also examined the vertical accuracy in localizing the relevant bar (when it was presented during the saccade). Confusing the bars’ identities is expected to cause a lot of variability as well as a systematic compression to the mean of the indicated vertical locations. Note that simply confusing the identities of the bars will not give rise to any systematic difference between the conditions, but it will give rise to an additional compression of indicated positions.

### Experiment 2

The methods of experiment 2 were very similar to those of experiment 1. Below, we only describe aspects of the methods in which experiment 2 differed from experiment 1.

#### Participants

Eight participants (age 30±6 years; four female) volunteered to take part in this experiment. Seven of them were right-handed and one left-handed. None of them had taken part in the pilot experiment and four of them had taken part in experiment 1, but were still naïve with respect to the aim of the study.

#### Procedure

**Fig 2:**
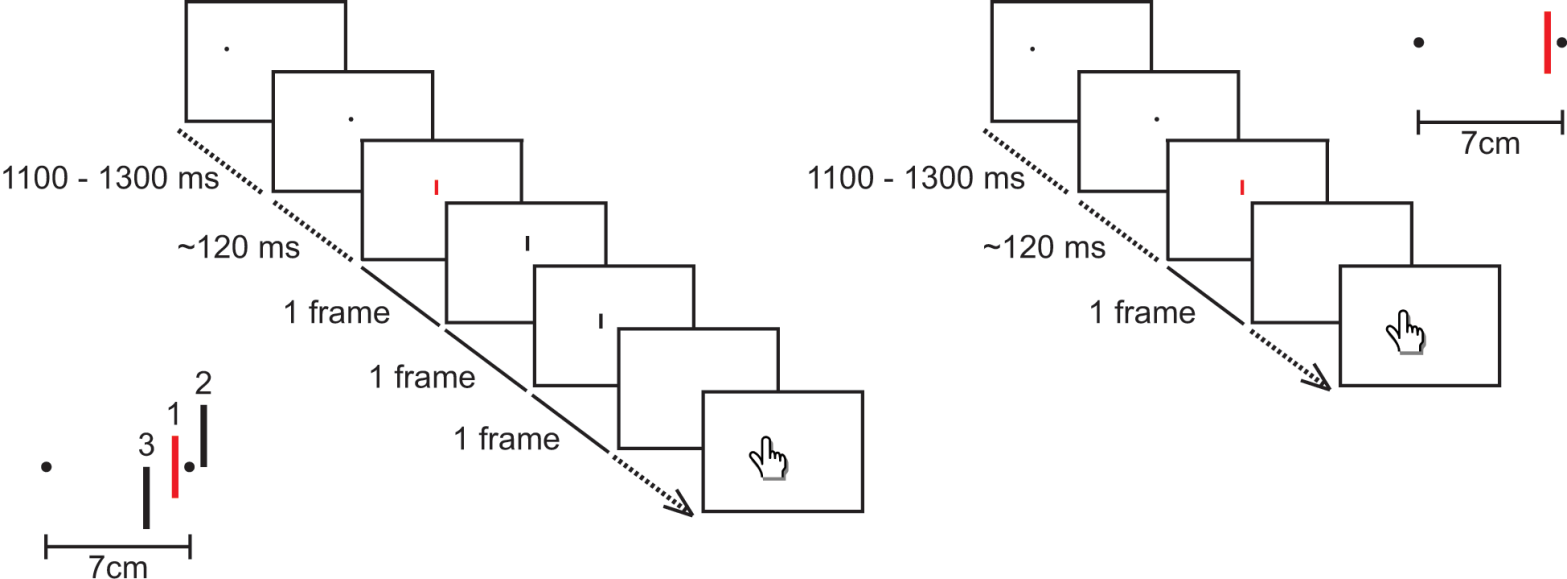
Schematic overview of two example trials in Experiment 2. The left part shows a trial in which two black bars were presented after the presentation of the red one. The inset shows the spatial configuration of this trial, with numbers indicating the order of presentation. The right part shows a trial in which only one red bar was presented. Details as in Fig 1.

Participants were asked to fixate a dot on the screen (Fig 2) and make a saccade from the fixation dot to the target dot as soon as the target dot appeared on the screen. Around the time of the saccade we presented a red bar for one frame, at one of the same five horizontal locations as in experiment 1. In half of the trials only the red bar was presented, always centered at the same vertical location as the fixation and target dots. In the other half of the trials, two black bars were presented after the presentation of the red bar (10 ms intervals). The two black bars were presented 1.4 cm to the left and right of the red bar. The three bars were always presented at different vertical locations. Their centres were at the same height as and 0.75 cm above and below the fixation and target dots, in random order.

There were 10 conditions in total: a single red bar or a red bar followed by two black bars, each with 5 horizontal locations of the red bar. Each session consisted of 400 trials; 40 for each of these conditions. All participants performed at least 3 sessions. On average, participants performed 4 sessions to yield reliable data in the relevant time-window. The four participants who had also taken part in experiment 1 performed fewer sessions in experiment 2 because we were able to present more flashes within the correct time window since we knew the characteristics of their saccades from experiment 1.

#### Data analysis

*Gaze and touch position*: Trials were discarded if there was no saccade between 100 ms before and 100 ms after the time of the presentation of the red bar (wrong timing; about 11% of the trials), if the length of the saccade was less than 50% or more than 150% of the 7 cm distance between the fixation and the target dot (wrong amplitude; about 7% of the trials), if the direction of the saccade deviated by more than 22.5^o^ from a movement to the right (wrong direction; about 3% of the trials), if the saccadic reaction time was less than 85 ms or more than 300 ms (wrong latency; about 7% of the trials) or if the touched location differed by more than 7 cm horizontally or by more than 3 cm vertically from the centre of the bar (wrong or no localization; mainly trials in which participants touched the bottom of the screen to indicate that they had not seen the red bar; about 1% of the trials).

As participants can only confuse the bars’ identities in trials with more than one bar, confusing the identity of the bars would lead to an increase in the vertical variability in trials with three bars. We therefore tested whether the vertical standard deviation in tapped location for the red flashes at the central vertical location was larger for trials with three bars than for trials with one bar using a one-tailed t-test.

## Results

### Pilot experiment

Participants reported the correct position in 24% of the trials, a position one item earlier or later in 39% of the trials, a position two items earlier or later in 23% of the trials, a position three items earlier or later in 12% of the trials, and a position four items earlier or later in 2% of the trials, which is only slightly better than chance (20%, 32%, 24%, 16% and 8%, respectively). Thus, the resolution of human temporal order judgments is indeed too poor to accurately identify the red bar’s temporal position in the sequence. Experiment 1 and 2 will capitalise on this finding by using sequences of flashes to influencing the perceived timing of a flashed red bar.

### Experiment 1

#### Eye movements

In total, 14552 useful localization judgments were obtained (about 76% of the 19200 trials); 7564 (about 79% of the 9600 trials) when the red bar was the second in the sequence and 6988 (about 73% of the 9600 trials) when the red bar was the fourth in the sequence. When the red bar was the second in the sequence the mean saccadic amplitude was 6.2 cm, the mean saccadic duration was 33 ms, the mean saccadic reaction time was 135 ms and the number of trials in which participants indicated that they had not seen the red bar was 320 (about 3.3% of the trials). When the red bar was the fourth in the sequence the mean saccadic amplitude was also 6.2 cm, the mean saccadic duration was 33 ms, the mean saccadic reaction time was 134 ms and the number of trials in which participants indicated that they had not seen the red bar was 255 (about 2.7% of the trials). The target dot had always disappeared by the time the saccade ended. Paired-samples t-tests showed no significant differences between the saccadic amplitudes, durations and reaction times for the two different orders of the red bar (all p>.05).

#### Mislocalization pattern

**Fig 3:**
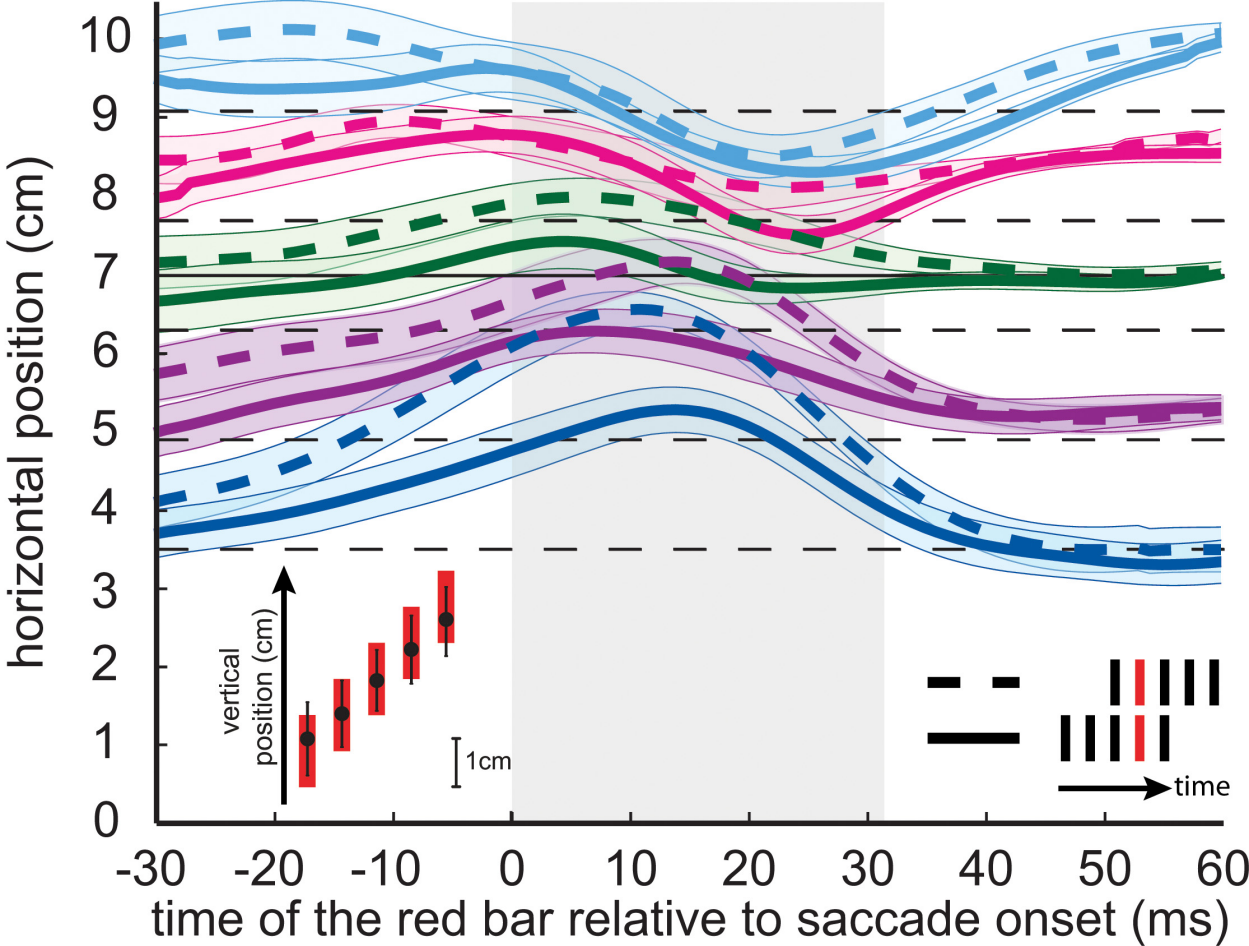
Average mislocalization curves in Experiment 1. Each thick curve is a smoothed average of the perceived locations of the red bar as a function of its time relative to saccade onset. The transparent areas surrounding these curves indicate the between-subjects standard error of the mean. The horizontal locations of the bars are indicated by dashed thin lines. Subjects made saccades from a fixation dot (at 0 cm) to a target dot (continuous thin line at 7 cm). The grey area shows the average saccade duration. The inset at the lower left shows that subjects were accurate in identifying the red bar: they indicated the vertical location accurately (symbols and error bars indicate the means and standard deviations of the indicated vertical locations when the red bar was presented at each of the five vertical locations that we used, only considering trials in which the red bar was presented during the saccade).

The pattern of mislocalization (Fig 3) is similar to the one found in previous studies, with a compression of perceived locations during the saccade and peaks in the mislocalization that occurred slightly earlier in the saccade for flashes that are closer to the fixation dot than for ones that are further away in the direction of the saccade target [8,13,19,20,43–45]. Most importantly, the position in the sequence of five bars clearly influenced the localization of the red bar: the dashed curves are systematically further in the direction of the saccade than the solid curves of the same colour, as we had predicted. This difference was 0.43 cm for the planned comparison during the saccade (one-tailed, one-sample t-test, p < .05). The inset in the lower left part of Fig 3 shows that participants had no problem isolating the red bar, as they quite accurately indicated its vertical location when it was presented during the saccade. We cannot judge whether the variability (and a slight compression towards the mean) is partly due to confusing some bars. In experiment 2 we will be able to do so.

### Experiment 2

#### Eye movements

In total, 8236 trials yielded useful localization judgments (about 71% of the 11600 trials); 3871 (about 67% of the 5800) trials in which only the red bar was presented and 4365 (about 75% of the 5800) trials in which two black bars were presented after the red bar. In trials in which only the red bar was presented, the mean saccadic amplitude was 6.3 cm, the mean saccadic duration was 32 ms, the mean saccadic reaction time was 132 ms and the number of trials in which participants indicated that they had not seen the red bar was 34 (about 0.6% of the trials). In trials in which two black bars were presented after the red bar, the mean saccadic amplitude was 6.3 cm, the mean saccadic duration was 31 ms, the mean saccadic reaction time was 132 ms and the number of trials in which participants indicated that they had not seen the red bar was 82 (about 1.4% of the trials). The target dot had always disappeared by the time the saccade ended. Paired-samples t-tests showed no significant differences between the saccadic amplitudes and reaction times for the two different orders of the red bar, but a significant difference between the saccadic durations in the two conditions (t(7) = 2.715, p < .05).

#### Mislocalization pattern

**Fig 4:**
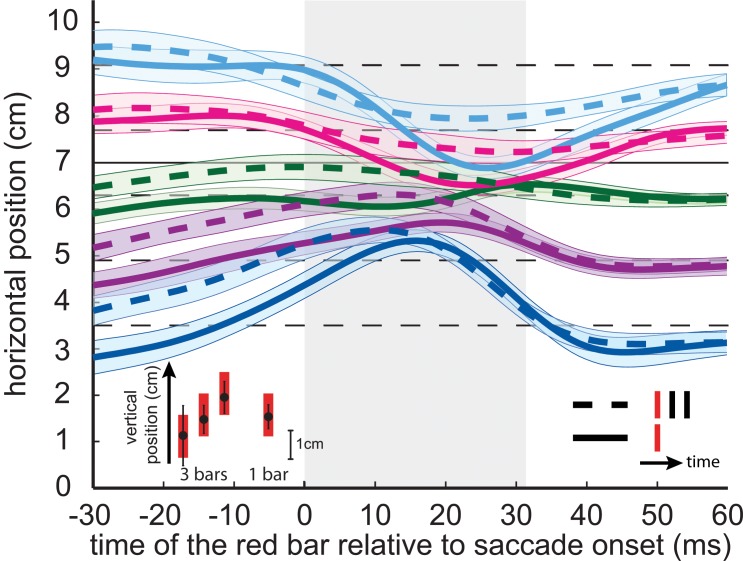
Average mislocalization curves in Experiment 2. Details as in Fig 3. The open symbols in the inset at the lower left indicate the vertical locations separately for the conditions with and without black bars, respectively.

For both conditions, the pattern of mislocalization (Fig 4) was similar to the one found in previous studies. Most importantly, where subjects indicated that they had seen the red bar was clearly influenced by presenting two black bars after the red one: dashed curves are systematically above the solid curves of the same colour, as predicted. Thus, the red bar was perceived to be further in the direction of the saccade when it was followed by two black bars. This difference was 0.37 cm for the planned comparison during the saccade (one tailed, one-sample t-test, p < .05). Even when the red bar was presented during the saccade, participants indicated its vertical location quite accurately (inset in the lower-left corner of Fig 4). Most importantly, the standard deviation in vertical location was not significantly larger when two black bars were flashed in addition to the red one (p = .074), showing that participants were able to isolate the red bar from the black ones.

## Discussion

Timing is obviously important when localizing a briefly presented stimulus around the time of a saccade. To localize the stimulus, information about its retinal location needs to be combined with information about the orientation of the eyes [5,9]. As the orientation of the eyes changes rapidly due to the saccade, even a small temporal mismatch between the two time-varying signals (retinal and oculomotor) will give rise to a systematic shift in the flashes’ apparent locations in the direction of the saccade [1–3,5,9,14–16]. In addition to such overall shifts of the whole visual scene, a compression of the flashes’ apparent locations towards the saccade target location [6–8,12,17,18] or towards the location that is fixated after the saccade [13,19,20] has also been found.

The cause of the compression is still under debate. Some believe that a combination of shifts and deformations in the brain’s representation of space accounts for the perisaccadic mislocalization. In that case, the temporary remapping of locations in space during the eye movement should affect all elements in a scene [7], before the stimulus of interest has been selected, leaving ordinal relationships between locations intact. Others believe that vision is a more active process [27]. In that case, localization depends critically on when the item of interest is considered to have been flashed; it is first detected and then the relevant eye orientation is estimated in order to localize it.

In order to shed light onto this debate, we conducted two experiments. In experiment 1, we examined whether presenting four irrelevant black bars, either predominantly before or predominantly after the relevant red one, influences the localization of the red bar. The red bar was presented second or fourth in the sequence. The total duration of the presentation of the stimuli was always the same (five bars at 10 ms intervals); only the timing of the red bar within the sequence switched. We hypothesized that the resolution of human temporal order judgments would be too poor to accurately identify the red bar’s temporal order in the sequence [22,34–38], which we verified for our stimuli in the pilot experiment. We expected participants’ judgments of the red bar’s time to therefore be shifted towards the centre of the sequence; the time of the presentation of the third bar.

We reasoned that if visual and oculomotor signals are combined at an earlier stage of processing than the selection of the red bar as the item of which the location is to be judged, then the mislocalization of the red bar would only depend on the time-dependent distortion of visual spatial information by signals related to the saccade. In that case, the time course of spatial mislocalization should be the same when the red bar is presented second as when it is presented fourth. In contrast, if the visual and oculomotor signals are combined after the red bar is selected, then misjudging the time at which the red bar was presented would result in the wrong eye orientation being used to estimate the red bar’s location. In that case, the temporal order of the red bar would affect its perceived location: if the red bar was presented second in the sequence it would be perceived as having occurred later in time, and thus be judged to be further in the direction of the saccade than if it was presented fourth in the sequence. The latter, which is in line with our prediction, was clearly the case.

If participants had not misjudged the timing of the red bar but had confused its identity with that of the other four bars, the judged locations would have been compressed towards the mean position of the bars. This would have yielded the same systematic effect for the two temporal positions of the red bar within the sequence, because the positions were randomized. Most importantly, participants correctly indicated the different vertical locations of the red bars even when they were presented during the saccade (inset in Figs 3 and 4), confirming that it must have been the time and not the identity of the red bar that was misjudged.

Despite the short time intervals between the bars, we cannot be completely certain that the red bar in experiment 1 was not processed faster when it was presented fourth than when it was presented second due to its appearance being cued earlier (because there were more preceding black bars). Moreover, the first black bar will more often have been presented before the eyes started to move when the red bar was presented as the fourth in the sequence than when it was presented second, so if the red bar is attracted towards the location of the bar presented before the saccade rather than towards the saccade target when such a bar is present [42], we would also find an average shift in the direction that we predicted (because the possible bar locations are not arranged symmetrically around the location of the saccade target). To address these concerns, we performed experiment 2, in which a single red bar was compared with a red bar followed by two black bars, so in none of the trials was a black bar presented before the red bar.

The results of experiment 2 showed a similar systematic influence of the black bars on perisaccadic localization of the red bar (Fig 4), with the perceived location of the red bar being further in the direction of the saccade when two black bars were presented after the presentation of the red bar, than when only the red bar was presented. This can be explained using the same reasoning as for experiment 1: by assuming that when two black bars followed the red one, participants’ judgments of the red bar’s time were shifted towards the centre of the sequence. This cannot be interpreted in terms of cueing, because there was no visible difference between the conditions until after the red bar had been presented. Thus, the pattern of perisaccadic mislocalization that we found in experiment 1 was probably not due to the preceding black bars speeding up the processing of the red bar, but to an effect of the time of the whole sequence of five bars on the perceived time of the red bar.

The results reported in the present study support the idea that the spatial mislocalization of stimuli flashed around the time of a saccade is not a consequence of time-dependent distortions of visual spatial information by signals related to the saccade, but arises from having to judge when the stimulus of interest was flashed with respect to the saccade. We conclude that the spatial localization of flashed stimuli involves judging the eye orientation at the estimated time of the flash.

## Supporting Information

S1 Appendix Appendix for Dataset of Experiment 1. (PDF)

S2 Appendix Appendix for Dataset of Experiment 2. (PDF)

S1 Dataset Dataset of Experiment 1, Participant 1. (ZIP)

S2 Dataset Dataset of Experiment 1, Participant 2. (ZIP)

S3 Dataset Dataset of Experiment 1, Participant 3. (ZIP)

S4 Dataset Dataset of Experiment 1, Participant 4. (ZIP)

S5 Dataset Dataset of Experiment 1, Participant 5. (ZIP)

S6 Dataset Dataset of Experiment 1, Participant 6. (ZIP)

S7 Dataset Dataset of Experiment 1, Participant 7. (ZIP)

S8 Dataset Dataset of Experiment 1, Participant 8. (ZIP)

S9 Dataset Dataset of Experiment 1, Participant 9. (ZIP)

S10 Dataset Dataset of Experiment 2, Participant 1. (ZIP)

S11 Dataset Dataset of Experiment 2, Participant 2. (ZIP)

S12 Dataset Dataset of Experiment 2, Participant 3. (ZIP)

S13 Dataset Dataset of Experiment 2, Participant 4. (ZIP)

S14 Dataset Dataset of Experiment 2, Participant 5. (ZIP)

S15 Dataset Dataset of Experiment 2, Participant 6. (ZIP)

S16 Dataset Dataset of Experiment 2, Participant 7. (ZIP)

S17 Dataset Dataset of Experiment 2, Participant 8. (ZIP)

## References

[1] Matin L, Pearce DG. Visual perception of direction for stimuli flashed during voluntary saccadic eye movements. Science 1965;148:1485–1488. 17738160 10.1126/science.148.3676.1485

[2] Bischof N, Kramer E. Investigations and considerations of directional perception during voluntary saccadic eye movements. Psychol Forschung. 1968;32:185–218. 10.1007/BF00418660 5715146

[3] Mateeff S. Saccadic eye movements and localization of visual stimuli. Percept Psychophys. 1978;24:215–224. 704282 10.3758/bf03206092

[4] Honda H. Perceptual localization of visual stimuli flashed during saccades. Percept Psychophys. 1989;45:162–174. 2928078 10.3758/bf03208051

[5] Dassonville P, Schlag J, Schlag-Rey M. Occulomotor localization relies on a damped representation of saccadic eye displacement in human and nonhuman primates. Vis Neurosci. 1992;9(3–4):261–269. 1390386 10.1017/s0952523800010671

[6] Morrone MC, Ross J, Burr DC. Apparent position of visual targets during real and simulated saccadic eye movements. J Neurosci. 1997;17:7941–7953. 9315912 10.1523/JNEUROSCI.17-20-07941.1997 PMC6793897

[7] Ross J, Morrone MC, Burr DC. Compression of visual space before saccades. Nature 1997;386:598–601. 9121581 10.1038/386598a0

[8] Lappe M, Awater H, Krekelberg B. Postsaccadic visual references generate presaccadic compression of space. Nature 2000;403:892–895. 10706286 10.1038/35002588

[9] Schlag J, Schlag-Rey M. Through the eye, slowly: Delays and localization errors in the visual system. Nat Rev Neurosci. 2002;3:191–215. 11994751 10.1038/nrn750

[10] Pola J. Models of the mechanism underlying perceived location of a perisaccadic flash. Vision Res. 2004;44:2799–2813. 15342224 10.1016/j.visres.2004.06.008

[11] Brenner E, Meijer WJ, Cornelissen FW. Judging relative positions across saccades. Vision Res. 2005;45:1587–602. 15781075 10.1016/j.visres.2004.11.020

[12] Maij F, Brenner E, Smeets JBJ. Temporal information can influence spatial localization. J Neurophysiol. 2009;102:490–495. 10.1152/jn.91253.2008 19439670

[13] Matziridi M, Brenner E, Smeets JBJ. Is mislocalization during saccades related to the position of the saccade target within the image or to the gaze position at the end of the saccade? PLoS One 2013;8(4):e62436 10.1371/journal.pone.0062436 23626819 PMC3633890

[14] Matin L, Matin E, Pola J. Visual perception of direction when voluntary saccades occur .2. Relation of visual direction of a fixation target extinguished before a saccade to a subsequent test flash presented before saccade. Percept Psychophys. 1970;8,9–14.

[15] Honda H. The extraretinal signal from the pursuit-eye-movement system: its role in the perceptual and the egocentric localization systems. Percept Psychophys. 1990;48:509–15. 2247334 10.3758/bf03211595

[16] Honda H. The time courses of visual mislocalization and of extraretinal eye position signals at the time of vertical saccades. Vision Res. 1991;31:1915–21. 1771775 10.1016/0042-6989(91)90186-9

[17] Honda H. Saccade-contingent displacement of the apparent position of visual stimuli flashed on a dimly illuminated structured background. Vision Res. 1993;33(5–6):709–16. 8351842 10.1016/0042-6989(93)90190-8

[18] Awater H, Burr DC, Goldberg ME, Lappe M, Morrone C. Perisaccadic compression after saccade adaptation. Perception 2001;30:24–24.

[19] Awater H, Lappe M. Perception of visual space at the time of pro- and anti-saccades. J Neurophysiol. 2004;91:2457–2464. 14973312 10.1152/jn.00821.2003

[20] Maij F, Brenner E, Smeets JBJ. Temporal uncertainty separates flashes from their background during saccades. J Neurosci. 2011;31:3708–3711. 10.1523/JNEUROSCI.5097-10.2011 21389225 PMC6622777

[21] Ross J, Morrone MC, Goldberg ME, Burr DC. Changes in visual perception at the time of saccades. Trends Neurosci. 2001;24:113–121. 11164942 10.1016/s0166-2236(00)01685-4

[22] Brenner E, van Beers RJ, Rotman G, Smeets JBJ. The role of uncertainty in the systematic spatial mislocalization of moving objects. J Exp Psychol Human 2006;32:811–825. 10.1037/0096-1523.32.4.811 16846281

[23] Brenner E, Mamassian P, Smeets JBJ. If I saw it, it probably wasn't far from where I was looking. J Vis. 2008;8:7,1–10. 10.1167/8.2.7 18318633

[24] Duhamel JR, Colby CL, Goldberg ME. The updating of the representation of visual space in parietal cortex by intended eye movements. Science 1992;255:90–92. 1553535 10.1126/science.1553535

[25] Nakamura K, Colby CL. Updating of the visual representation in monkey striate and extrastriate cortex during saccades. Proc Natl Acad Sci USA 2002;99:4026–4031. 11904446 10.1073/pnas.052379899 PMC122642

[26] Kusunoki M, Goldberg ME. The time course of perisaccadic receptive field shifts in the lateral intraparietal area of the monkey. J. Neurophysiol. 2003;89:1519–1527. 12612015 10.1152/jn.00519.2002

[27] O’Regan JK, Noë A. A sensorimotor account of vision and visual consciousness. Behav Brain Sci. 2001;24:939–1031. 12239892 10.1017/s0140525x01000115

[28] Brenner E, Smeets JBJ. Motion extrapolation is not responsible for the flash-lag effect. Vision Res. 2000;40:1645–1648. 10814752 10.1016/s0042-6989(00)00067-5

[29] Lappe M, Kuhlmann S, Oerke B, Kaiser M. The fate of object features during perisaccadic mislocalization. J Vis. 2006;6:1282–1293. 17209735 10.1167/6.11.11

[30] Ernst M, Banks M. Humans integrate visual and haptic information in a statistically optimal fashion. Nature 2002;415:429–433. 11807554 10.1038/415429a

[31] Alais D, Burr DC. The ventriloquist effect results from near-optimal bimodal integration. Curr Biol. 2004;14:257–262. 14761661 10.1016/j.cub.2004.01.029

[32] Binda P, Bruno A, Burr DC, Morrone MC. Fusion of visual and auditory stimuli during saccades: A Bayesian explanation for perisaccadic distortions. J Neurosci. 2007;27:8525–8532. 17687030 10.1523/JNEUROSCI.0737-07.2007 PMC6672948

[33] Binda P, Morrone MC, Burr DC. Temporal auditory capture does not affect the time course of saccadic mislocalization of visual stimuli. J Vis. 2010;10(2):7,1–13. 10.1167/10.2.7 20462308

[34] Battelli L, Walsh V, Pascual-Leone A, Cavanagh P. The ‘when’ parietal pathway explored by lesion studies. Curr Opin Neurobiol. 2008;18:120–126. 10.1016/j.conb.2008.08.004 18708141 PMC5076376

[35] Nava E, Bottari D, Zampini M, Pavani F. Visual temporal order judgment in profoundly deaf individuals. Exp Brain Res. 2008;190:179–188. 10.1007/s00221-008-1459-9 18563399

[36] Linares D, Holcombe AO, White AL. Where is the moving object now? Judgments of instantaneous position show poor temporal precision (SD = 70 ms). J Vis. 2009;9(13):9,1–14. 10.1167/9.13.9 20055542

[37] van Mierlo CM, Louw S, Smeets JBJ, Brenner E. Slant cues are processed with different latencies for the online control of movement. J Vis. 2009;9(3):25,1–8. 10.1167/9.3.25 19757964

[38] Brenner E, Smeets JBJ. How well can people judge when something happened? Vision Res. 2010;50:1101–1108. 10.1016/j.visres.2010.03.004 20214919

[39] Brainard DH. The psychophysics toolbox. Spatial Vision 1997;10:433–436. 9176952

[40] Cornelissen FW, Peters EM, Palmer J. The Eyelink Toolbox: Eye tracking with MATLAB and the Psychophysics Toolbox. Behav Res Meth Instr. 2002;34:613–617. 10.3758/bf03195489 12564564

[41] Maij F, Brenner E, Li HC, Cornelissen FW, Smeets JBJ. The use of the saccade target as a visual reference when localizing flashes during saccades. J Vis. 2010;10(4):7,1–9. 10.1167/10.4.7 20465327

[42] Cicchini GM, Binda P, Burr DC, Morrone MC. Transient spatiotopic integration across saccadic eye movements mediates visual stability. J Neurophysiol. 2013;109: 1117–1125. 10.1152/jn.00478.2012 23197453 PMC3569120

[43] Awater H, Lappe M. Mislocalization of perceived saccade target position induced by peri-saccadic visual stimulation. J Neurosci. 2006;26:12–20. 16399668 10.1523/JNEUROSCI.2407-05.2006 PMC6674306

[44] Ostendorf F, Fischer C, Finke C, Ploner CJ. Perisaccadic compression correlates with saccadic peak velocity: differential association of eye movement dynamics with perceptual mislocalization patterns. J Neurosci. 2007;27:7559–63. 17626217 10.1523/JNEUROSCI.2074-07.2007 PMC6672618

[45] Maij F, Brenner E, Smeets JBJ. Peri-saccadic mislocalization is not influenced by the predictability of the saccade target location. Vision Res. 2011;51:154–159. 10.1016/j.visres.2010.10.024 21035479

