# Supplementary material for: The Role of Temporal Information in Perisaccadic Mislocalization
Source: PLoS One. 2015 Sep 9;10(9):e0134081. doi: 10.1371/journal.pone.0134081 (PMC4564215; doi:10.1371/journal.pone.0134081)
Supplement: S1 Appendix — (PDF) [file pone.0134081.s001.pdf]

MAIN EXPERIMENT

DATA FROM THE .mat FILES:

LOCATIONS OF STIMULI ON THE SCREEN (in pixels):

xy___ = location (x,y) of the fixation dot
xy____ = location (x,y) of the saccade target dot
bar1_xy_ = coordinates (x1,y1,x2,y2) of the 1st bar
bar2_xy_ = coordinates (x1,y1,x2,y2) of the 2nd bar
bar3_xy_ = coordinates (x1,y1,x2,y2) of the 3rd bar
bar4_xy_ = coordinates (x1,y1,x2,y2) of the 4th bar
bar5_xy_ = coordinates (x1,y1,x2,y2) of the 5th bar
bar_list_(:,6) = order of the red bar in the sequence of five bars (2nd or 4th)
mouse_location_x = touch location (x) on the screen
mouse_location_y = touch location (y) on the screen
ppd = pixels per degree

TIIMG OF THE PRESENTATION OF THE STIMULI (in ms):

dot1_presentation = presentation time of the fixation dot
dot2_presentation = presentation time of the saccade target dot
flash1_time_displ_ = presentation time of the 1st bar
flash2_time_displ_ = presentation time of the 2nd bar
flash3_time_displ_ = presentation time of the 3rd bar
flash4_time_displ_ = presentation time of the 4th bar
flash5_time_displ_ = presentation time of the 5th bar
exafanish_olwn = disappearance of all stimuli
time_delay = time of the presentation of the red bar (synchronized with the eye movement recordings)

DATA FROM THE .eps FILES:

eye_used = eye used for the analysis (2 = right eye)
data.FSAMPLE.gx(eye_used,:) = gaze (x) coordinates
data.FSAMPLE.gy(eye_used,:) = gaze (y) coordinates
data.FSAMPLE.time = gaze time

If more information is needed, don't hesitate to contact me to mmatziridi@yahoo.gr
