# Supplementary material for: The Role of Temporal Information in Perisaccadic Mislocalization
Source: PLoS One. 2015 Sep 9;10(9):e0134081. doi: 10.1371/journal.pone.0134081 (PMC4564215; doi:10.1371/journal.pone.0134081)
Supplement: S2 Appendix — (PDF) [file pone.0134081.s002.pdf]

CONTROL EXPERIMENT

DATA FROM THE .mat FILES:

LOCATIONS OF STIMULI ON THE SCREEN (in pixels):

bar_all_(:,20:21) = location (x,y) of the fixation dot
bar_all_(:,22:23) = location (x,y) of the saccade target dot
bar_all_(:, 1:4) = coordinates (x1,y1,x2,y2) of the 1st (red) bar
bar_all_(:, 12:15) = coordinates (x1,y1,x2,y2) of the 2nd bar (if 3 bars are presented on the screen, otherwise 0)
bar_all_(:, 16:19) = coordinates (x1,y1,x2,y2) of the 3rd bar (if 3 bars are presented on the screen, otherwise 0)
bar_all_(:,5) = number of bars presented in each trial (1 or 3)
mouse_location_x = touch location (x) on the screen
mouse_location_y = touch location (y) on the screen
ppd = pixels per degree

TIIMG OF THE PRESENTATION OF THE STIMULI (in ms):

dot1_presentation = presentation time of the fixation dot
dot2_presentation = presentation time of the saccade target dot
flash1_time_displ_ = presentation time of the 1st (red) bar
oo = 1 frame
flash1_time_displ_ + oo = presentation time of the 2nd bar (only if condition with 3 bars, otherwise ignore it)
flash1_time_displ_ + oo + oo = presentation time of the 3rd bar (only if condition with 3 bars, otherwise ignore it)
exafanish_olwn = disappearance of all stimuli
time_delay = time of the presentation of the red bar (synchronized with the eye movement recordings)

DATA FROM THE .eps FILES:

eye_used = eye used for the analysis (2 = right eye)
data.FSAMPLE.gx(eye_used,:) = gaze (x) coordinates
data.FSAMPLE.gy(eye_used,:) = gaze (y) coordinates
data.FSAMPLE.time = gaze time

If more information is needed, don’t hesitate to contact me to mmatziridi@yahoo.gr
